# αSiC - βSiC - graphene composites

**DOI:** 10.1038/s41598-023-31539-2

**Published:** 2023-03-15

**Authors:** Ali Razmjoo, Hamid Reza Baharvandi, Nasser Ehsani

**Affiliations:** Faculty of Composite Materials, Malek Ashtar University of Technology, Tehran, Iran

**Keywords:** Mechanical and structural properties and devices, Nanoparticles, Composites

## Abstract

In this study, the mechanical properties of the pressureless sintered samples of α-SiC based composite containing 0–3 wt.% graphene and 0–15 wt.% nano β-SiC were investigated. Simultaneous usage of nano β-SiC and graphene and transformation of β-SiC (3C) to α-SiC (6H/4H) resulted in elongation of secondary α-SiC grains, which significantly improved the mechanical properties (e.g. fracture toughness) of SiC ceramics. According to the results, the highest Relative density of 99.04%, Young’s modulus of 537.76 GPa and fracture toughness of 5.73 MPa × m^1/2^ were obtained in the sample containing 5 wt.% nano β-SiC and 1 wt.% graphene (5B1G). In addition, two methods of measuring bending strength including three-point bending tests and biaxial tests (piston-on-three-ball) were compared. Strip-shaped specimens were prepared for three-point bending test and disc-shaped specimens were prepared for biaxial bending test. Each bending test was evaluated using a universal testing machine. The results showed that the biaxial bending strength is less than the three-point bending strength. Also, the maximum three-point bending strength of 582.01 MPa and biaxial bending of 441.56 MPa were obtained in 5 wt.% Nano β-SiC and 1 wt.% Graphene samples (5B1G). Studies have shown that in addition to the many advantages of using the biaxial bending method, the results have a very similar trend to the three-point bending strength. Also, the most-increased hardnesses of 28.03 GPa and 29.97 GPa were seen in the sample containing 5 wt.% nano β-SiC (5B) with forces of 10 N and 1 N, respectively. One of the effective mechanisms in improving the fracture toughness of α-SiC ceramics is crack deflection/bridging. Also, the difference in thermal expansion of the α-SiC matrix and the reinforcements, leading to the creation of residual stresses between the matrix grains and the reinforcement, is effective in improving the mechanical properties (e.g. strength and fracture toughness).

## Introduction

Nowadays, silicon carbide (SiC) is a widely used non-oxide ceramic with a global production rate of about 700,000 tons/year. Due to its ultra-high hardness and heat/oxidation resistance, it is employed as an abrasive and raw material for producing parts such as refractory furnaces and heating elements^1–9^. SiC has two different crystal structures β-SiC and α-SiC with more than 180 polytypes. The 3C polytype with cubic structure is known as β-SiC and another polytypes (Hexagonal and Rhombohedral) are known as α-SiC. 6H, 4H, and 2H are the most common α-SiC polytypes. At high temperatures, β-SiC (3C) is unstable and transforms to α-SiC (6H/4H), leading to an increase in grain length^8,10,11^. Another important application of SiC is its usage as a siliconizing and carburizing agent in iron and steel metallurgy. However, the applications of SiC due to its low fracture toughness and poor sintering are limited, and therefore many studies have already been conducted in this area^11–16^.

Additives and high temperatures are required for SiC sintering^11,17^. Depending on the type and amount of additive, SiC ceramics can be compacted by sintering in the solid or liquid state^18–24^. Solid-state sintering usually requires a sintering temperature above 2100 °C^25–27^. Sintering additives achieve high density by reducing grain boundary energy and reacting with the remained silica in SiC particle surfaces^9,11^. In contrast, the liquid-state sintering process performed at temperatures between 1850 and 2000 °C degrades some properties such as the high-temperature fracture toughness^28–36^. In recent years, using nanotechnology to improve the properties of SiC ceramics has been attracting attention. Accordingly, the use of nanoparticles as a reinforcement compared to micro-sized ones has resulted in more noticeable properties^37,38^.

Among the various additives that have been used so far, graphene, due to its superior strength, thermal/electrical conductivity, and mechanical strength, can be a suitable additive to improve the properties of SiC ceramics^8,39–43^. High specific surface area is another unique advantage of graphene. All chemical reactions/interactions are performed on the surface of nanomaterials, and therefore the specific surface area is of great importance in determining the reactivity of materials. The specific surface area of graphene is calculated to be 2630 square meters per gram, while one gram of carbon nanotubes has a surface area of 500 square meters^8^. Li et al.^44^ and Guo et al.^45^ are among the researchers who have studied the effect of graphene addition on the properties of pressureless-sintered SiC ceramics, which will be briefly discussed in the following.

Li et al.^44^ investigated the effect of different amounts of graphene (0–5 wt.%) along with 1 wt.% B_4_C on SiC ceramics pressureless sintered at 2130 °C. According to the results of this study, the hardness of 29 GPa, fracture toughness of 5.65 MPa × m^1/2^, and Bending strength of 420 MPa in the sample containing 1 wt.% graphene were obtained. Moreover, owing to exceptional thermal conductivity and high electron mobility in graphene, the thermal conductivity of SiC samples was enhanced by increasing graphene content from 0 to 2 wt.%. Meanwhile, the carbon existing in the grain boundary prevents the growth of SiC grains, which can have a significant effect on fracture toughness.

In another study, Guo et al.^45^ sintered SiC ceramics along with graphene and B_4_C additives by pressureless sintering technique at 2200 °C. According to the findings, adding 1 wt.% graphene resulted in relative density, Bending strength, and hardness of 99%, 367 MPa, and 22 GPa, respectively. The authors found out that the low thermal expansion coefficient of graphene reduces the shrinkage of SiC ceramics.

Moreover the deflection and distortion of graphene layers, which is probably due to compression during the compression and sintering process, can increase the bending strength and fracture toughness by wasting some of the fracture energy, as it can prolong the fracture transmission path, thus wastes more fracture energy^39^.

Today, biaxial bending tests are mainly performed by the International Organization for Standardization (ISO) to evaluate the bending strength of dental ceramic materials^46^. However, few studies of biaxial bending tests have been performed on SiC ceramics^47^. Biaxial testing has been claimed to further explore the limits of strength to brittle materials by testing larger volumes than beam bending. Preparing disc or square parts, especially in the laboratory, is easier than rod test parts and requires less machining. In addition, edge preparation is less important because the maximum stresses applied are far from the edges^46^. However, there are factors such as sample fabrication methods, test protocols, or material strength that must be considered when measuring bending strength. In addition, the stress distribution in the specimens may affect the strength due to the brittleness of the ceramic material. It is known that it is very difficult to prevent the effect of the edge of the shape on the rectangular specimens^46^. Biaxial bending tests provide reliable values because the maximum stress is at the center of the specimen and the effect of holes and cracks on the edges can be reduced^48^. There is a report that the biaxial bending test method is more reliable for ceramics than the three-point and four-point bending test, because the Weibull modulus in the biaxial bending test was larger than other bending test methods^49^. However, there are few reports comparing the two bending test methods for SiC ceramics. In this research, for the first time, biaxial bending tests along with three-point bending tests were used to evaluate the bending strength of SiC ceramics with the addition of nano β-SiC and graphene.

Also, active mechanisms in improving the fracture toughness of SiC ceramics reinforced by graphene include crack deflection, crack bridging, tearing-open regions, relative slip between graphene layers, and crack branching^8^. In addition, the transformation of β-SiC (3C) to α-SiC (6H/4H) generates longer, interlocking grains that tend to increase the SiC fracture toughness^10,23,50–53^.

According to the previous research works, mainly the effect of the addition of graphene along with B_4_C on the properties of SiC ceramics has already been studied^8^. Also, β-SiC has been mainly used as the matrix phase, which was difficult to achieve high density due to the poor sinterability of β-SiC. In addition, previous research showed that the presence of large amounts of β-SiC increases porosity and decreases properties^10,23,50–53^.

In this research, for the first time, nano β-SiC was used as an additive along with graphene, and the effects of β-SiC to α-SiC transformation on mechanical properties (e.g. fracture toughness) were investigated. The results showed that the elongated secondary α-SiC grains can prevent the excessive growth of the grains and thus increase the density. Moreover, these grains can increase the fracture toughness by mechanisms such as crack deflection.

## Experimental procedure

### Starting materials and fabrication method

**Figure 1:**
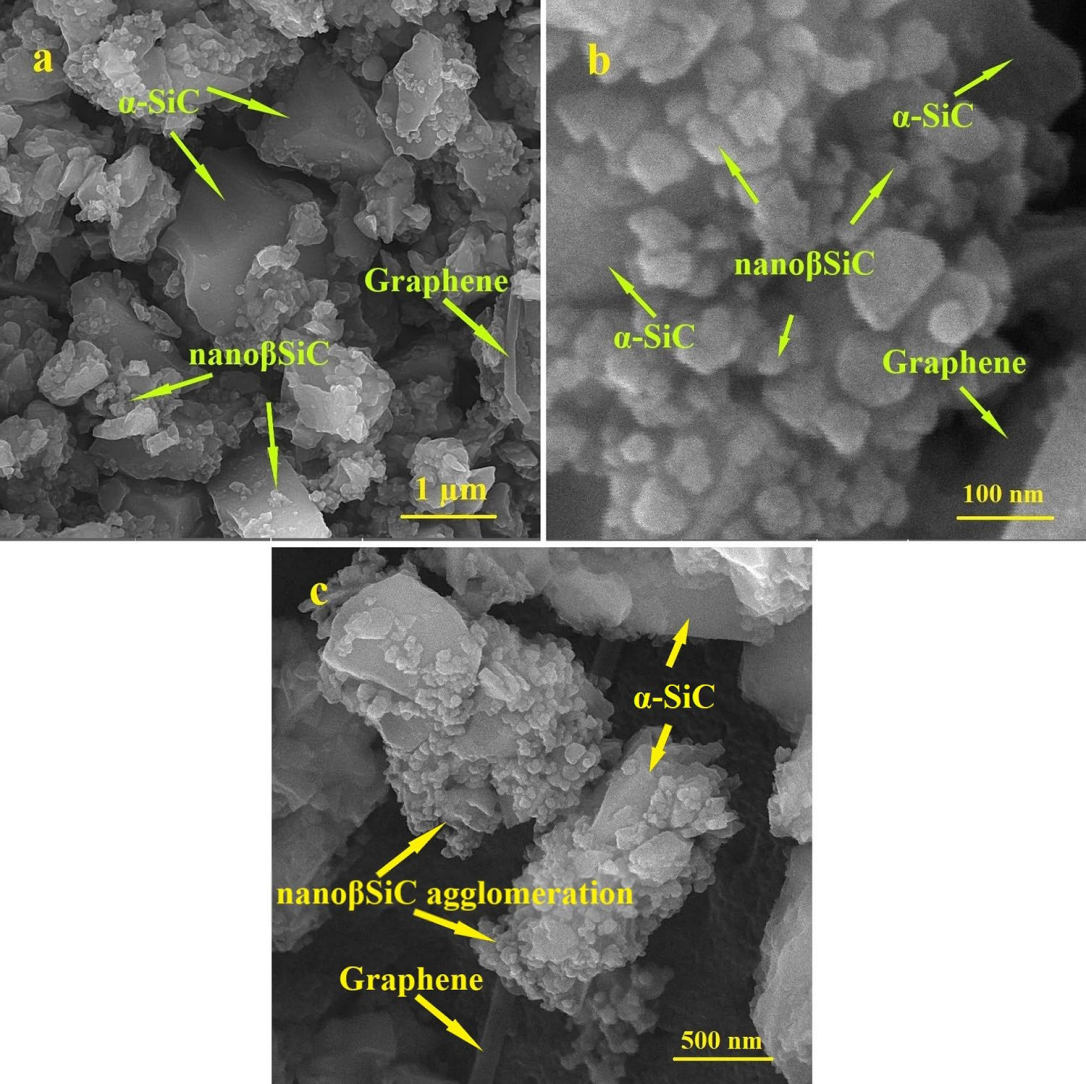
FESEM images of samples (**a**,**b**) 5B1G in two magnifications and (**c**) 15B3G after 3 hours of grinding.

In this study, α-SiC powder with an average submicron size of particles and a purity of 99%, was used as the main powder. α-SiC powder mainly contains 6H polytype. β-SiC nanopowder with 99% purity and an average particle size of 50 nm, including majorly 3C polytype, was used as an additive. Also, multilayer graphene powder with 99% purity, with an average particle size of 5–10 microns, layer thickness of 4–20 nm, and the number of layers less than 20 were used as additives. The starting composition and labels of the prepared samples are given in Table 1. The precursors were first ground in a planetary ball mill with WC balls and ethanol solution as the milling medium for 3 h at a speed of 180 rpm. FESEM images of samples 5B1G (at two magnifications) and 15B3G after 3 hours of grinding are shown in Fig. 1a–c, respectively.

**Table 1:** Weight percentage of each material and the sample codes.

Sample code	Nano β-SiC (wt.%)	Graphene (wt.%)	α-SiC (wt.%)
1	0	0	Balance
5B	5	0	Balance
10B	10	0	Balance
15B	15	0	Balance
1G	0	1	Balance
5B1G	5	1	Balance
10B1G	10	1	Balance
15B1G	15	1	Balance
2G	0	2	Balance
5B2G	5	2	Balance
10B2G	10	2	Balance
15B2G	15	2	Balance
3G	0	3	Balance
5B3G	5	3	Balance
10B3G	10	3	Balance
15B3G	15	3	Balance

The compounds were then dried at 90 °C for 12 h. The as-milled samples were first compressed under uniaxial compression (75 MPa) and then through the CIP process under a pressure of 150 MPa. In order to remove volatile organic chemicals or compounds, all samples were pyrolyzed up to 800 °C at a heating rate of 2 °C/min and finally sintered in argon atmosphere at 2200 °C for 2 h. After the end of the process, the furnace was turned off and the samples were slowly cooled to room temperature. It should be noted that for better distribution of nano β-SiC and graphene and to prevent the agglomeration of particles in the matrix, the ultrasonic bath process was used.

### Material characterization

In this research, the density and porosity of the samples were determined using the Archimedes’ principle by the immersion method in deionized water according to the ASTM C373 standard.

The phase analysis of the samples was conducted using an Inel EQUINOX 3000 instrument equipped with a Cu cathode and the microstructure of the samples was characterized by a field emission electron microscope (FESEM) (Tescan model) equipped with an energy dispersive spectroscopy (EDS) analyzer.

The X-ray diffraction (XRD) measurements were performed for crystalline phases analysis of the samples using a Philips diffractometer instrument in the range of 10°–80° equipped with a copper Ka1 radiation (0.15406 nm wavelength) source and a nickel filter. The obtained XRD patterns were reorganized by X’Pert High Score software.

A Takram, Teksan apparatus (laser wavelength of λ = 532 nm, magnification of 50 × and power of 0.5–70 mW) was used for Raman microscopy and at least 3 scans were performed for each sample.

The mean grain size of the long edge length using 200 grains was determined by using MIP software based on SEM images of the different etched areas at the same magnification.

### Mechanical characterization

To evaluate the effect of adding graphene along with nano β-SiC on the properties of SiC ceramics, a set of tests was used to measure Young’s modulus, Hardness, fracture toughness and bending strength (three-point and biaxial).

The Young’s modulus (E) of the specimens was determined using the ultrasonic method by computing the changes in sound velocity in the specimen (based on the ASTM C769 standard), according to Eq. (1).

1 $$ {\text{E }} = \, \rho {\text{V}}^{{2}} , $$

where ρ is its density (Kg/m^3^) and V is the speed of sound (m/s) across the sample.

The Vickers hardness of the samples was determined by Hardness Instrument (Koopa hardness evaluating device, UV1 model) using a 9.8 N load (ASTM C1327 standard). At least five well-defined indentations were considered on each sample and the average of the measured values was reported. The Vickers hardness was computed from Eq. (2).

2 $$ H = (1.8544)\frac{P}{{d^{2} }}, $$

where P is the applied load (N) and d is the diagonal of the indentation mark (mm).

The Fracture toughness (*KIC*) was calculated by the Eq. (3), according to ANSTIS^54^ and NIIHARA^55^, calculations.

3 $$ K_{IC} = a\left( \frac{E}{H} \right)^{0.5} \left( {\frac{P}{{c^{3/2} }}} \right), $$

where α is the indenter geometry constant for Vickers diamond pyramid indenter with the amount of 0.016 ± 0.004, E is the Young’s modulus (GPa), H is the Hardness (GPa), P is the applied force to put on Vickers effect, and c is the crack length (mm) from the center of the indent to the crack tip. The applied load was also 9.8 N with a load time of 30 s.

The Bending strength (modulus of rupture : MOR) was determined by three point Bending tests using an outer span of 10 mm and a displacement rate of 0.5 mm/min, and using small bars of 25.0 mm × 4.0 mm × 3 mm. The Bending strength was then calculated by the following equations according to ASTM C1161 standard:

4 $$ MOR = \frac{3Fl}{{2bd^{2} }}, $$

where F is the fracture force (N), l is the distance between the supporting columns (mm), b is the width of sample (mm) and d is the thickness of sample (mm), respectively. The Bending strength measurements were performed on 4 samples.

The Piston-on-three-ball test was used to evaluate the Biaxial bending of ceramic specimens following the ISO 6872 specifications. The piston was flat-ended with a diameter of 1.4 mm. The three supporting balls were 3.2 mm in diameter and positioned apart at an angle of 120 degrees on a support circle with a diameter of 10 mm. The specimens were positioned on the three supporting balls concentrically and loaded by the flat-ended piston with a crosshead speed of 1 mm/min using a universal testing machine (Instron 3345, Electromechanical, Norwood, MA, USA). To reduce the friction a polyethylene sheet was placed between the specimen and the piston.

The biaxial bending was calculated as follows:

5 $$ \sigma \, = \, - 0.{\text{2387 P }}\left( {{\text{X }} - {\text{ Y}}} \right)/{\text{b}}^{{2}} , $$

σ is the Biaxial bending (MPa), P is the total load causing fracture (N),

6 $$ {\text{X }} = \, \left( {{1 } + {\text{ n}}} \right){\text{ ln }}\left( {{\text{B}}/{\text{C}}} \right)^{{2}} + \, \left[ {\left( {{1 } - {\text{ n}}} \right)/{2}} \right] \, \left( {{\text{B}}/{\text{C}}} \right)^{{2}} , $$

7 $$ {\text{Y }} = \, \left( {{1 } + {\text{ n}}} \right) \, \left[ {{\text{l }} + {\text{ ln }}\left( {{\text{A}}/{\text{C}}} \right)^{{2}} } \right] \, + \, \left( {{1 } - {\text{ n}}} \right) \, \left( {{\text{A}}/{\text{C}}} \right)^{{2}} , $$

n = Poisson’s ratio (If the value for the ceramic concerned is not known, use n = 0.25), A = radius of supporting circle (mm), B = radius of loaded area (mm), C = radius of specimens (mm), b = specimen thickness at fracture origin (mm).

### Ethical approval

Based on the research and observations of the Faculty of Composites, the tests performed in this study do not have a negative effect on human tissue.

## Results and discussion

### Phase analysis

**Figure 2:**
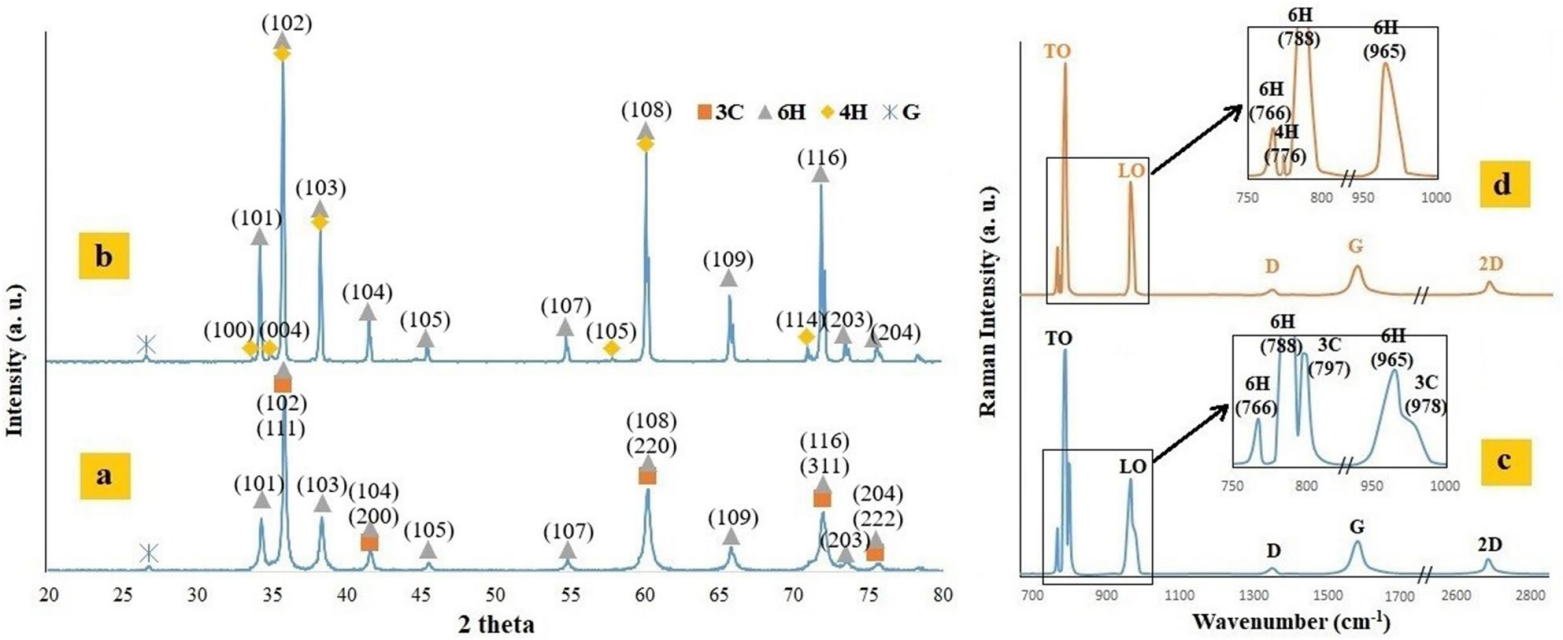
The XRD patterns of sample 5B1G, (**a**) before and (**b**) after the sintering process and The Raman spectroscopy of sample 5B1G, (**c**) before and (**d**) after the sintering process.

The XRD patterns of sample 5B1G before and after the sintering process are displayed in Fig. 2a and b respectively. As shown in Fig. 2a, the major polytypes are 6H and 3C along with graphene. However, according to Fig. 2b, after the sintering process, there is no trace of 3C polytype and some 4H polytype is formed. It seems that due to the high sintering temperature and the instability of 3C polytype, the transformation of nano β-SiC particles to α-SiC has occurred. This transformation leads to elongation of SiC grains and production of secondary α-SiC^8,10,11^.

Due to the high interference of 6H and 3C peaks in the XRD pattern, Raman spectroscopy of sample 5B1G before and after the sintering process (Fig. 2c,d) was used for better distinguish and display of polytypes. Bands transverse optic (TO) and longitudinal optic (LO) were identified as coming from silicon carbide. As shown in Fig. 2c, before the sintering process, the TO peak region contains wavenumbers 766, 788, and 797 cm^−1^, representing 6H, 6H and 3C, respectively. In addition, the LO peak region contains wavenumbers 965 and 978 cm^−1^, representing 6H and 3C, respectively^56–59^. However, according to Fig. 2d, after the sintering process, there is no trace of 3C polytype that confirms the XRD results.

**Figure 3:**
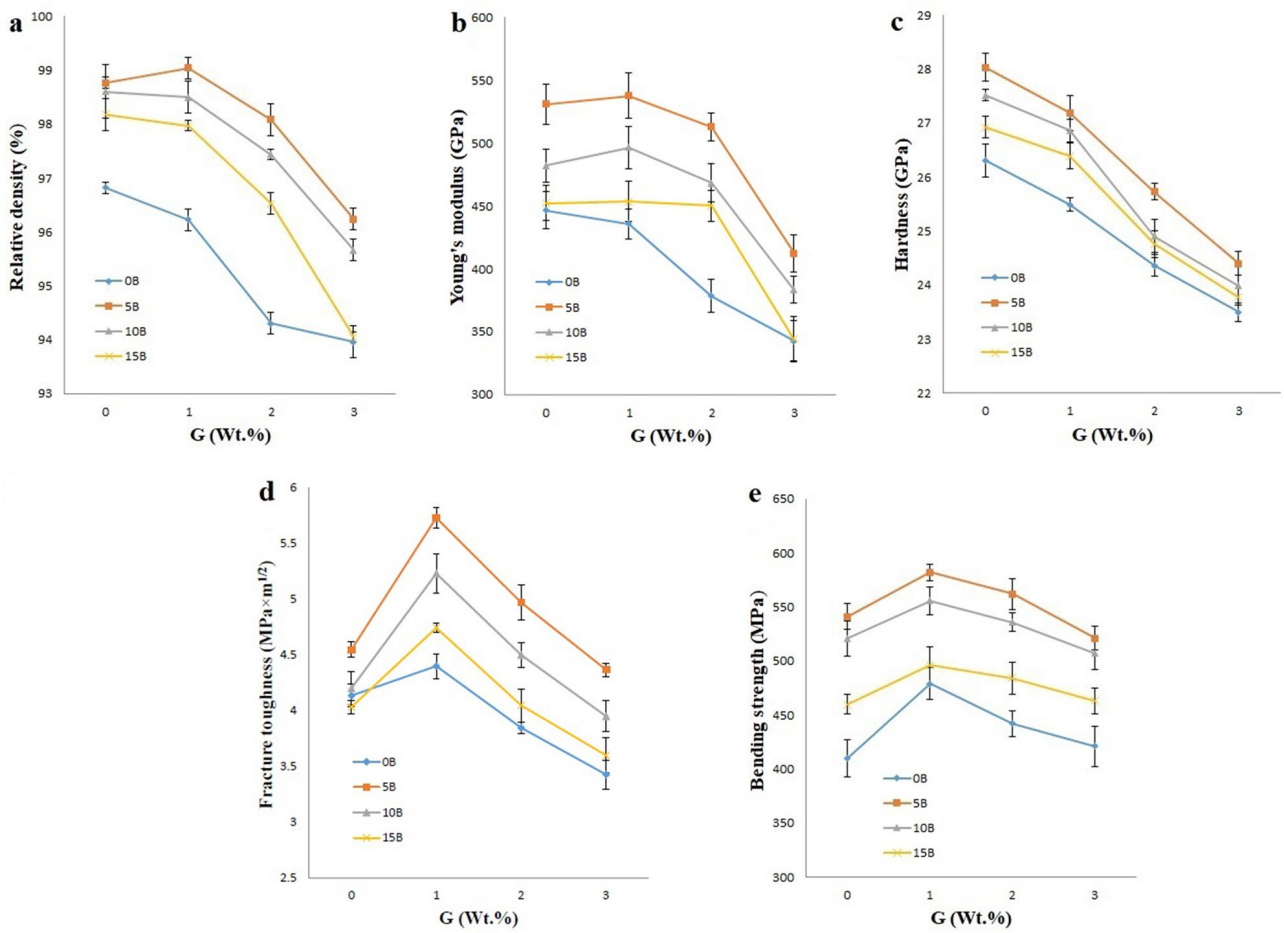
Changes in (**a**) Relative density, (**b**) Young’s modulus, (**c**) Hardness, (**d**) Fracture toughness and (**e**) Three-point Bending strength with different percentages of graphene and nano β-SiC.

In addition, the carbon bands (D, G and 2D) that represent the graphene are visible in Fig. 2c,d, indicating that the structure of graphene does not change. Moreover, Raman spectra for sample 5B1G shows the 2D-band (Fig. 2c,d), which is typical for multilayer graphene. The stability of the graphene structure can help increase the final strength of the specimens^8,60^. In the following, the results of relative density, Young’s modulus, hardness, toughness and three-point bending strength are shown in Fig. 3a–e, respectively.

### Relative density and microstructure

**Figure 4:**
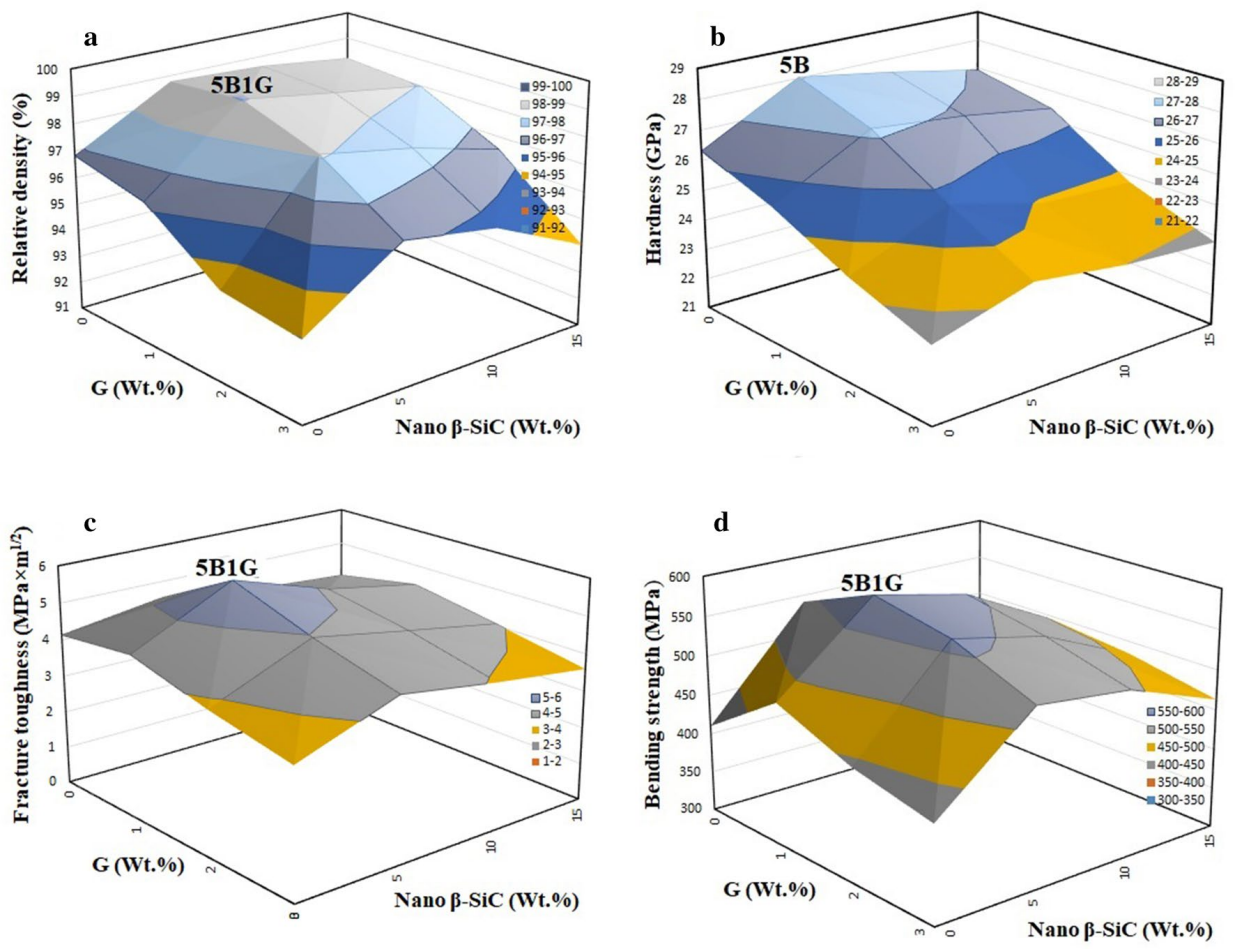
Three-dimensional surfaces for (**a**) Relative density, (**b**) Hardness, (**c**) Fracture toughness and (**d**) Three-point Bending strength, of SiC composite samples as a function of both different amounts of graphene and nano β-SiC.

The Relative density results for all sinters are presented in Fig. 3a. Almost all samples show a considerable decrease of Relative density alongside with the increase of graphene content. One of the reasons for this reduction can be the improper distribution and the presence of porosity in high amounts of graphene layers as an additive. However, according to the results, the highest relative density (99.04%) was obtained in the optimum sample (5B1G). In addition, the three-dimensional surface of the Relative density changes of SiC composite specimens as a function of graphene and nano β-SiC is illustrated in Fig. 4a. According to Fig. 4a, sample 5B1G is located at the highest point of the surface and the density decreases with changes in the amounts of graphene and nano β-SiC. Inducing the proper distribution of β-SiC nanoparticles along with the effect of graphene on inhibiting grain overgrowth in the sample could be one of the reasons for this optimal density.

Small amounts of graphene layers combined with nano β-SiC have a better distribution (Fig. 1a,b). Moreover, high amounts of additives (graphene layers and nano β-SiC) have a higher tendency to agglomerate (Fig. 1c). This phenomenon has a negative influence on the graphene dispersion in the matrix, causing higher porosity^60^.

In addition, the graphene layers can be considered as a C (carbon) source that can remove the SiO_2_ layers on the SiC surface and by reducing the ratio of the grain boundary energy to the surface energy can provide the driving force for the diffusional mass transport for sintering^8,10^.

**Figure 5:**
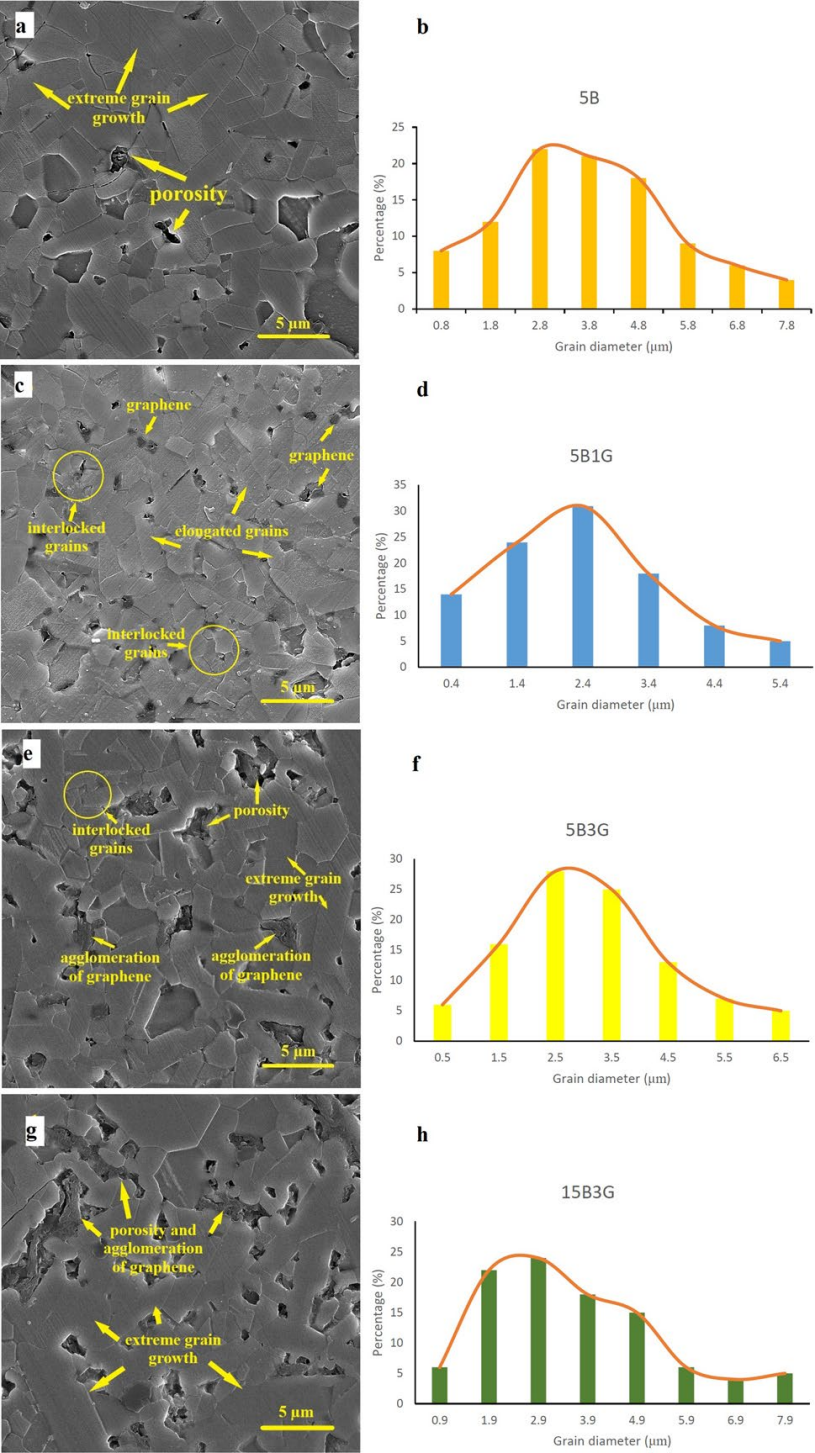
FESEM images of microstructures and grain size distributions of samples (**a**,**b**) 5B, (**c**,**d**) 5B1G, (**e**,**f**) 5B3G and (**g**,**h**) 15B3G.

In the following, as mentioned, to obtain the grain size, two linear intercept methods and the use of MIP software were used by measuring a large number of grains. Figure 5a–h shows the FESEM microstructures and grain size distributions of samples 5B, 5B1G, 5B3G and 15B3G respectively.

The microstructure of the four samples show significant differences. The microstructure of sample 5B is without graphene layers and includes grains with extreme growth along with some intergranular porosity (Fig. 5a,b). The microstructure of sample 5B1G mainly includes elongated grains with a relatively fine morphology (Fig. 5c,d). Also, proper distribution and small dimensions of graphene layers are well observed. According to the phase analysis, all nano β-SiC particles have been transformed into secondary α-SiC during the transformation process. It seems that grains with submicron dimensions and elongated shape are the same as secondary α-SiC. Compared to samples 5B, 5B3G and 15B3G, the grain size distribution of sample 5B1G is more uniform and finer. This can be proven by histograms. Also, the microstructure of sample 5B3G includes extreme growth grains (Fig. 5e,f). In addition, graphene layers are seen cumulatively next to each other along with pores. Next, the microstructure of sample 15B3G includes grains with extreme growth along with some intergranular porosity (Fig. 5g,h). The average sizes of samples 5B, 5B1G, 5B3G and 15B3G are approximately 3.74, 2.37, 3.14 and 3.63 µm, respectively, based on their grain size distribution histograms. This investigation shows that the grain size of the sintered samples is effectively reduced by the uniform distribution of the particles. Based on this, the 5B1G sample had the best distribution and the smallest grain size among the samples.

### Young’s modulus

The effects of different amounts of graphene and nano β-SiC on Young’s modulus of the samples are presented in Fig. 3b.

The results indicate that by increasing the weight percent of nano β-SiC particles to 5 wt.% and graphene to 1 wt.%, the Young’s modulus is increased to a maximum of 537.76 Gpa. While more increase in the amount of nano β-SiC caused a decrease in Young's modulus. It seems that the particle distribution in the samples containing up to 5 wt.% nano β-SiC is suitable (Fig. 1a,b and Fig. 5a,c). However, with increasing the amount of nano β-SiC (due to the extreme grain growth, which reduces the relative density and increases the porosity), Young’s modulus decreases. The extreme grain growth and porosity can be observed in the FESEM images of Fig. 5e,g, while the rate of porosity and extreme grain growth in Fig. 5c are lower compared to them. On the other hand, since the Young's modulus indicates the presence of strong bonds in the material, increasing the porosity reduces it^31,60^. Moreover, increasing graphene from 0 to 3 wt.% due to the accumulation of graphene layers and porosity increase causes the overall decrease in Young’s modulus of the samples (Fig. 5e,g). According to the results, the presence of porosity has reduced the sound velocity in the samples. Since the sound velocity is part of mechanical waves, its emission and transmission require material; therefore, the more the porosity and the lower the density of the material, the more sound velocity decreases^31,60^and according to Eq. (1), Young’s modulus is also reduced.

### Hardness

The effects of different amounts of graphene and nano β-SiC on the hardness of the sintered samples are shown in Fig. 3c. The load applied to measure the hardness in this step was 10 N. Based on the results, all samples show a significant decrease in Hardness with increasing graphene content.

In the following, the hardness changes of the SiC composite samples as a function of graphene and β-SiC nanoparticles are shown in three dimensions in Fig. 4b. The 5B sample is at the maximum surface point (28.03 GPa) and the hardness decreases with changes in graphene and nano β-SiC.

According to the results, in all samples, the hardness increased with increasing nano β-SiC up to 5 wt.%, but by using more nano β-SiC content, the hardness decreased. Factors affecting the hardness of sintered SiC ceramics include density, the grain size of the matrix, coefficient of thermal expansion of the matrix and reinforcements^9,11,31^. According to previous studies, increasing the density causes an increase in the hardness. Meanwhile, the presence of coaxial grains and fine-grained structure increases the hardness^60^. In samples containing nano-sized reinforcements, one of the important and influential factors on the mechanical properties can be the agglomeration phenomenon of reinforcing particles that control this phenomenon can have good impacts on the mechanical properties of the samples^8^. According to the results, by increasing the amount of nano β-SiC and graphene to higher than 5 and 1 wt.%, respectively, particle agglomeration and porosity are observed (Fig. 5g), which is one of the important factors affecting the hardness reduction of the samples. Moreover, in sample 5B, the absence of graphene reduced the agglomeration phenomenon and increased the hardness (Fig. 5a). Other factors affecting the hardness of the samples include the effect of adding reinforcements on the grain size of the ceramic matrix. According to FESEM images of samples containing different amounts of nano β-SiC and graphene additives, by increasing the amount of nano β-SiC and graphene to higher than 5 and 1 wt.%, respectively, grain size and porosity increase leading to a decrease in the hardness of the samples (Fig. 5g,h). Moreover, as seen in Fig. 3c due to the low hardness of graphene compared to that of SiC, the addition of graphene particles reduces the hardness of the samples, similar to previous works. Increasing porosity through increasing graphene content decreased the hardness as well^8^.

In addition, at high levels of β-SiC, one of the problems during sintering of SiC components is the transformation of β-SiC polytype to α-SiC, which causes extreme grain growth and subsequent increase in cavities between the particles through stacking fault (SF), which reduces the hardness of SiC parts^61^. This has not affected the hardness reduction of the sample containing 5 wt.% nano β-SiC due to its appropriate particle distribution and small amount. SiC sintering at ultra-high temperatures can lead to the transformation of the cubic polytype phase to hexagonal one. This phase transformation also occurs in the sample 5B1G. This is consistent with the presence of elongated grains next to the coaxial grains in the FESEM image of the sample 5B1G (Fig. 5c).

**Figure 6:**
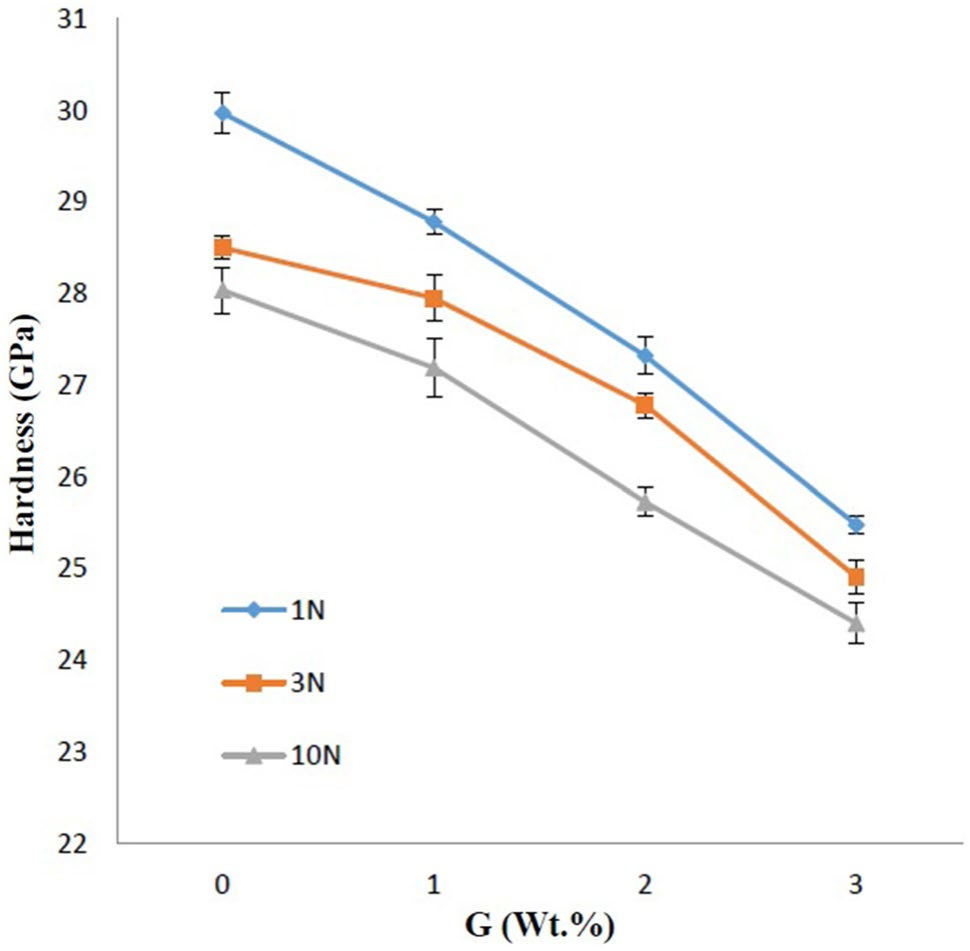
The effect of different loads on hardness.

In addition, two forces of 1 and 3 N were used to investigate the effect of changing the applied force on the hardness next to the 10 N load. The graph of these changes for samples containing 5 wt.% nano β-SiC with different graphenes is shown in Fig. 6. The changes indicate a decrease in hardness with increasing load, which is based on the elastic–plastic model of discrete deformed bonds in very hard materials including SiC^27^. According to this model, the size of the indentation effect is a combination of the effect of elastic recovery and plastic deformation. This model also assumes that the rate of elastic recovery at low indentation load is relatively higher than the formation of a new plastic region. Therefore, at lower loads, the dimensions of the effect are smaller and, as a result, the hardness is higher^27^. Based on the obtained results, the highest hardness of 29.97 GPa was obtained in sample 5B with a force of 1 N. Of course, it seems that applying more load due to the larger effect diameter covers a wider area of the material and is therefore closer to reality.

### Toughness

Figure 3d shows the effect of different amounts of graphene and nano β-SiC on the fracture toughness of sintered SiC ceramics. Also, the changes in fracture toughness with additives can be seen more comprehensively in the three-dimensional diagram by considering the different amounts of graphene and nano β-SiC additives (Fig. 4c).

As shown in Figs. 3d and 4c, by increasing the weight percent of nano β-SiC particles to 5 wt.% and graphene to 1 wt.%, the fracture toughness is increased to a maximum of 5.73 MPa × m^1/2^. With increasing the amount of additives in the 15B3G sample, the fracture toughness decreased as much as 3.59 MPa × m^1/2^. According to studies, fracture toughness is directly related to material density^60^. Grain size, porosity, and the obtained microstructure are important and influential factors affecting fracture toughness^36,37^. Elongation of the grains is also another effective factor in improving toughness^30,31^.

According to previous studies, transformation of 3C to 6H and 6H to 4H polytypes generate more elongated and interlocked grains, that tends to increase the fracture toughness of SiC. In addition, primary β-SiC (3C) can develop the columnar inter-grains of 4H phase as secondary α- SiC with relatively uniform composition and grain size distribution, which increases fracture toughness^23,50–53^. The elongated and interlocked grains are shown in Fig. 5c.

Indeed, the crack growth path is increased by elongating the matrix grains, which promotes the fracture toughness of SiC ceramics^11,27^. In this study, the simultaneous addition of graphene and nano β-SiC had a significant effect on the elongation of SiC grains. With a dense microstructure and a limited amount of porosity, elongated grains may act as a reinforcement, however, in the case of similar ceramic specimens, elongated grains do not perform this role by excessively increasing the porosity result in a lower amount of K_IC_^60^. This fact is observed in the 5B1G sample with very low porosity compared to other samples (Fig. 5c). Moreover, the presence of thin layers of graphene and their ultra-strong connection with the matrix cause its effective participation in the reinforcement of the material, which results in high amounts of K_IC_^40,60^. In addition, it is expected that the force required to pull out a graphene sheet be increased due to the large specific surface area and wrapping around the ceramic grains. Also, the relatively large particle size of graphene provides a long deflection path. These specifics contribute to enhancing the toughness of SiC/graphene composites and make graphene a good reinforcement^45^. Another issue effective in increasing the toughness is the pinning effect of graphenes as an obstacle to the growth of SiC grains, which in turn can modify the microstructure^8,40^. The pinning effect of graphenes as a barrier to the growth of SiC grains is clearly seen in Fig. 5c.

Also, the difference in the thermal expansion coefficient of graphene, SiC, and nano β-SiC can cause residual tensile stresses at the intergranular surfaces after sintering followed by cooling and therefore cause microcracks as the primary source of cracking. These microcracks scatter energy and hinder the growth of the main crack, eventually leading to enhanced fracture toughness. Since the coefficient of thermal expansion of graphene, αSiC, and β-SiC are respectively around − 4 × 10^–6^/°C, 4.5 × 10^–6^/°C, and 3 × 10^–6^/°C, tensile stress remains between the SiC and graphene grains after sintering. The presence of such stress may weaken the interphase boundaries but leads to the intergranular fracture^62^.

**Figure 7:**
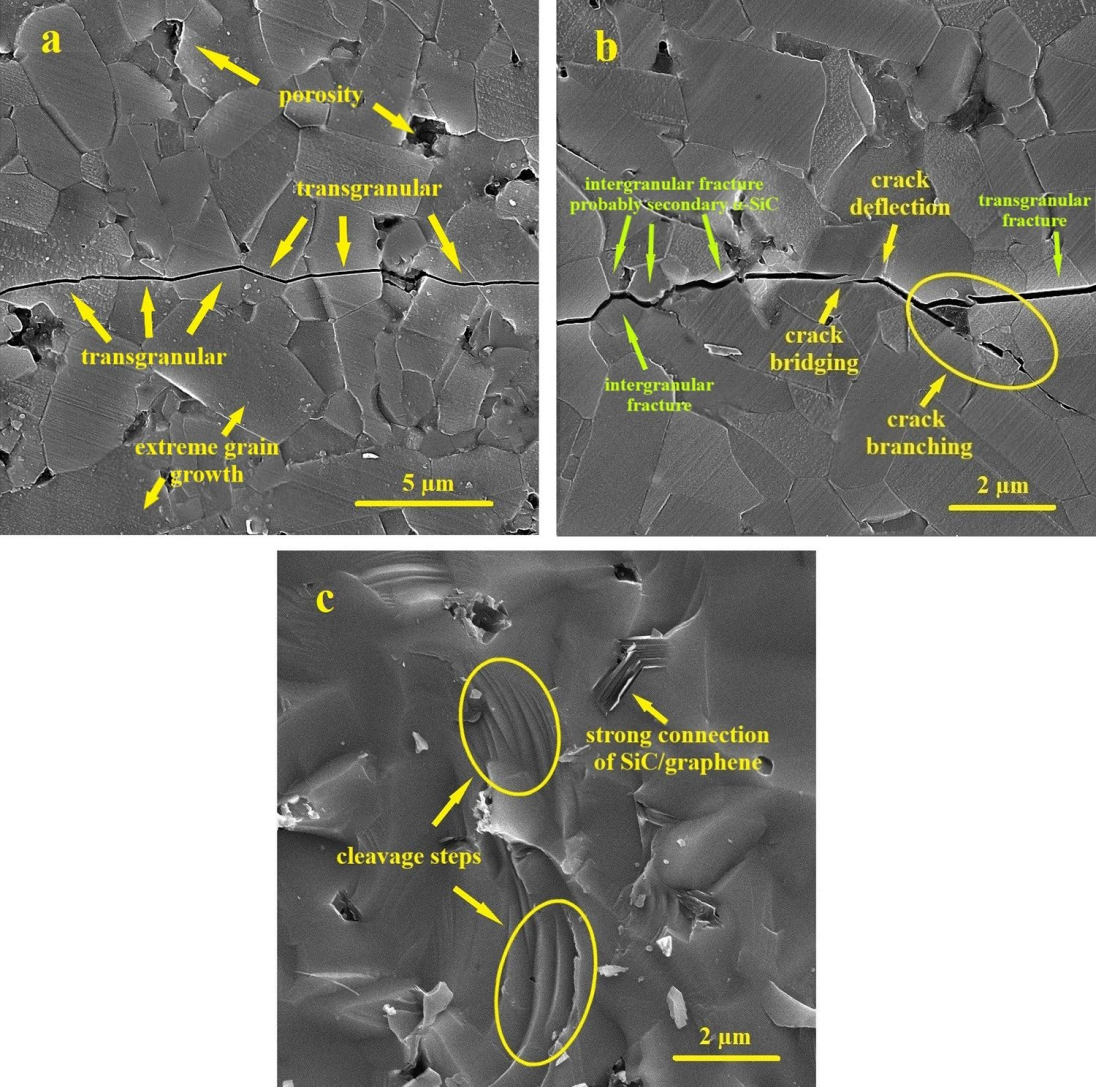
FESEM images of crack microstructure and its growth path in samples (**a**) 1 and (**b**) 5B1G, and (**c**) FESEM image of fracture surface in sample 5B1G.

Figure 7a,b show the FESEM micrographs of morphology and the crack path created by the Vickers Indenter for samples 1 (pure SiC) and 5B1G, respectively. As observed in Fig. 7b (sample 5B1G), regarding the proper distribution of reinforcements and proportional growth of matrix grains along with the transformation of nano β-SiC to α-SiC and elongation of these grains, mechanisms such as crack bridging and deflection increase the toughness^8,24,63^. On the other hand, the fracture toughness increases provided the particles with appropriate strength and density are well sintered. However, in the case of high porosity and low density, we will encounter a decrease in toughness. As observed in Fig. 5g,h, by increasing the weight percentages of nano β-SiC and graphene to higher than 5 and 1 wt.% respectively and consequently, agglomeration of nanoparticles as well as severe grain growth, the fracture toughness is reduced^8^.

Crack deviation, branching and bridging mechanisms are the most important and common mechanisms in improving the fracture toughness of solid-phase sintered SiC ceramics^64^. As seen in Fig. 7b, the presence of elongated grains along with coaxial ones plays an important role in improving the fracture toughness of SiC ceramics since the microstructures containing elongated grains have high fracture toughness. As mentioned, due to high temperature transformation, all β-SiC particles have been transformed to α-SiC. Although primary and secondary α-SiC grains are structurally identical and it is impossible to separate them in FESEM images, elongated grains with submicron dimensions can represent secondary α-SiC grains (Fig. 7b). According to research done on microstructures containing elongated and coaxial grains, high crack deflection is observed in the crack growth path, which affects crack energy reduction and improves the fracture toughness of SiC ceramics (Fig. 7b). On the other hand, the effect of graphene particles (as obstacles to crack growth) on the deviation of the crack growth path is prominent. The crack severely weakens when it strikes graphene layers in its growth path. On the other hand, due to the strong graphene/SiC interface, the crack does not have the ability to pull out and disperse particles, so it must be deflected from its original path. This crack deflection will consume a lot of energy and eventually increase the toughness^8,64^. In addition, since dislocations are formed around the reinforcing particles, if the crack encounters these dislocations in its growth path, it inevitably shows a path deviation^65,66^. Overall, these factors have improved the fracture toughness by increasing the reinforcing particles to 5 wt.% of nano β-SiC and 1 wt.% of graphene (Fig. 7b). Another reason for the decrease in the toughness of samples containing more than the optimal amount of reinforcements can be an excessive increase in residual tensile stress in the matrix phase, making it more vulnerable^67,68^.

In Fig. 7a, FESEM image of crack microstructure and its growth path of sample 1 (with no additive) is observed. As seen in Fig. 7a, in case that there are no reinforcements or additives in the structure, the crack growth path is mainly transgranular and almost in a straight line, and therefore the final fracture is mainly transgranular. However, by the addition of nano β-SiC and graphene particles, crack through deviation towards the grain boundary in its growth path overshadows more of them, and as a result, the percentage of intergranular grain boundary fracture increases^69^. In the 5B1G sample (Fig. 7b), the crack path occurs in a combination of both intergranular and transgranular modes, resulting in a more significant loss of energy from the crack.

In addition, the fracture surface analyses of the 5B1G sample using FESEM images are shown in Fig. 7c. As seen in Fig. 7c, the presence of stepped surfaces within the grains, which represent the cleavage steps, is quite evident, highlighted with ovals in the image. Stepped cleavage surfaces, like dislocations, can deviate the crack path^69^. It can also be seen in Fig. 7c that the fracture surfaces are wavy, indicating that the presence of reinforcing particles also affects the fracture surface morphology and causes its distortion. In addition, the release of residual stresses and the formation of frontal process zone (FPZ) also play an important role in improving toughness^67,68^.

**Figure 8:**
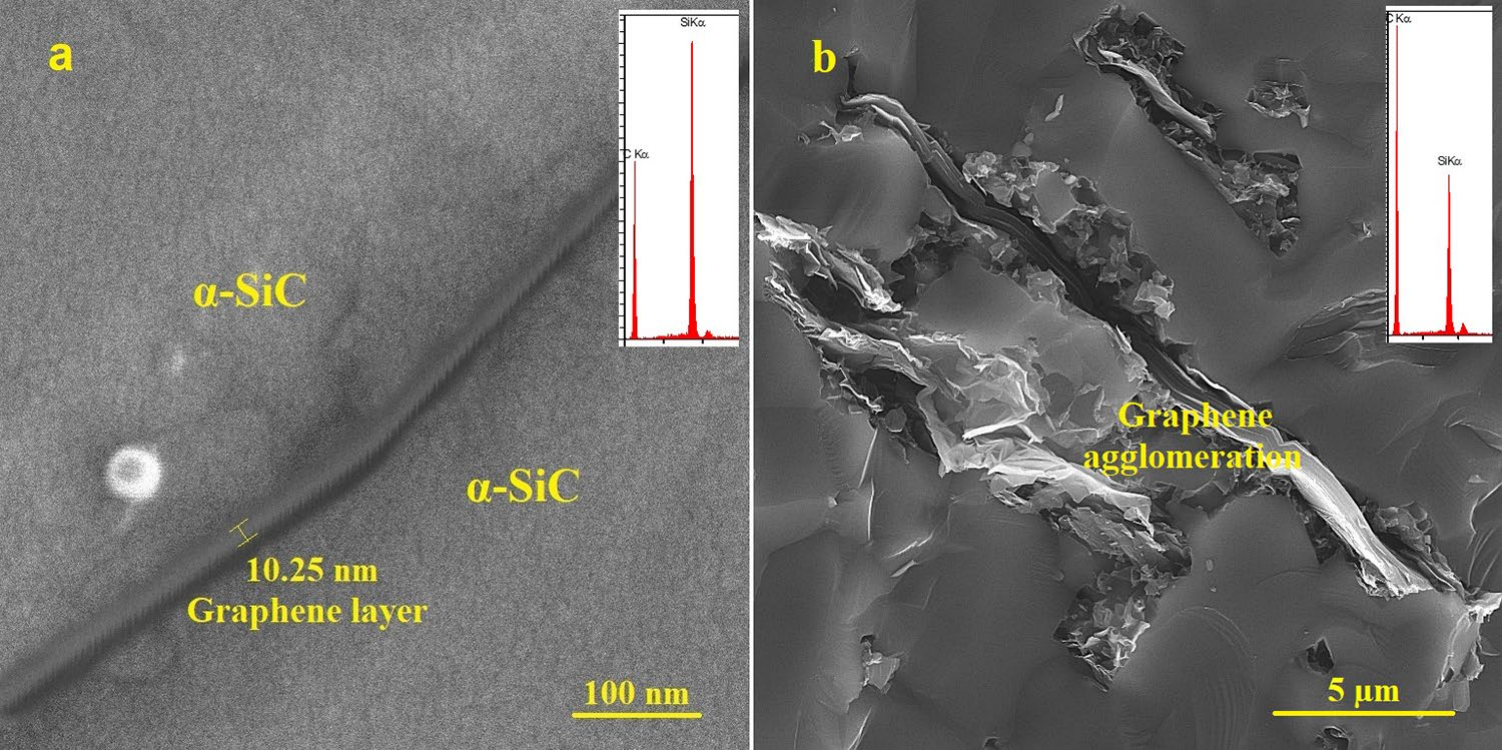
FESEM images of (**a**) 5B1G sample and connection of graphene layer with α-SiC matrix and (**b**) 10B3G sample and graphene agglomeration.

In order to thoroughly analyze the influence of graphene on the mechanical properties, a FESEM with EDS analysis of 5B1G and 10B3G specimens was performed. Figure 8a (5B1G) shows a layer of multilayer graphene (EDS confirms the existence of a graphene layer) strongly connected to the matrix, which confirms its effective participation in reinforcing the material and explains high values of mechanical properties (e.g. strength and fracture toughness). Based on the Fig. 8a Graphene has maintained its initial layered structure and had a proper distribution in the matrix. Further, in the Fig. 8b (10B3G) with increasing amount of graphene, agglomeration phenomenon has occurred and there is no effective connection between graphene and matrix. This problem reduces the mechanical properties.

### Bending strength

The Three-point bending strength of samples with different amounts of graphene and nano β-SiC are presented in Fig. 3e. The results show that the addition of nano β-SiC particles up to 5 wt.% and graphene up to 1 wt.%, increases the strength of the nanocomposite, while their higher amounts reduce the strength. In addition, changes in the Three-point bending strength of the specimens through the addition of reinforcing particles as a three-dimensional surface are observed in Fig. 4d and the sample 5B1G is at the maximum surface point of 582.01 MPa.

As the amount of graphene increases up to 1 wt.%, both the Three-point bending strength and the fracture toughness of the composites increase. The observed increase in Three-point bending strength and fracture toughness of SiC matrix composites is mainly attributed to the following reasons. First, the strong surface bond between SiC and graphene guarantees efficient charge transfer between the SiC matrix and graphene. Second, since the pinning effect of graphene inhibits the growth of SiC grains, the addition of graphene can modify the SiC grains to reinforce the SiC matrix. Third, graphenes can be used as a barrier to deflection, terminate, and bridging of cracks, which inhibit movements of dislocations, thus significantly improving the mechanical strength of the SiC composite^8,40,64^. Figure 7a,b, are FESEM micrographs of 1 (pure SiC) and 5B1G samples, respectively. From these two figures, it can be found that the grain size of the 5B1G sample is much smaller than that of sample 1 (pure SiC), indicating that fine-grain reinforcing has occurred in the 5B1G composite. Figure 7b shows crack deflection and crack branching occurred by graphenes. Good surface bonding between graphene and SiC is very important for enhancement mechanisms of strength and toughness. As graphene content increases more, Three-point bending strength and fracture toughness start to decrease. This is due to the formation of agglomerated graphenes, which causes large defects and weakens the effect of graphene (Fig. 5e,g).

Moreover, among the important and key factors in improving the Three-point bending strength of the specimens, we can mention the density, microstructure containing coaxial and elongated grains, as well as the difference in the coefficient of thermal expansion of the matrix and reinforcing phases^8,11,36^.

According to previous studies, Three-point bending strength of sintered SiC ceramics with graphene and other additives was about 200 to 620 MPa^8,39,40,43–45,70^. However, according to the results obtained from the Three-point bending strength test in this study (sample 5B1G showed the highest degree of Three-point bending strength of 582.01 MPa), the usefulness of these reinforcements in improving the Bending strength of SiC ceramics is proven. In the sample 5B1G, maximum strength is obtained, which can be due to the coherent distribution of particles and microstructure containing coaxial and elongated grains. The higher the volume fraction of the reinforcing particles, the greater the obstacles to the grain boundaries and, consequently, the less their mobility, provided we do not encounter an increase in the porosity and agglomeration of the particles. Inhibition of grain boundary mobility reduces grain size. According to Eq. (8) (known as Hall–Petch equation), grain size directly affects the strength of the material while reducing grain size increases the material strength. The relationship between yield stress (σ_y_) and grain diameter (d) or the Hall–Petch relationship^71^ is defined as Eq. (8).

8 $$ \sigma_{y} = \sigma_{0} + \frac{k}{\sqrt d }, $$

where σ_o_ and k are chemistry and microstructure dependent constants.

On the other hand, reinforcing particles placed in cavities and porosities reduce the size and density of structural defects that limit the strength of the material. Also, the mismatch between the thermal expansion of the matrix phase (SiC) and the reinforcing phase (graphene) causes residual stress in the structure, which increases with increasing the volume fraction of the reinforcing particles. The presence oweekf residual stress prevents crack nucleation and growth in the grain boundary, thus increasing the strength of SiC ceramics^8,36,39,40,64^. Decreased strength by adding more than 5 wt.% nano β-SiC can be attributed to particle agglomeration and consequently the formation of intergranular cavities, which are a good place for crack nucleation. Moreover, the addition of too much graphene and nano β-SiC increases the amount of residual stress in the structure. The high amount of residual stress causes the matrix phase (which is subject to endure the residual tensile stress) not to have the capacity to tolerate this level of stress, and cracks will be created inside it, and thus the matrix will fracture by applying less external stresses.

**Figure 9:**
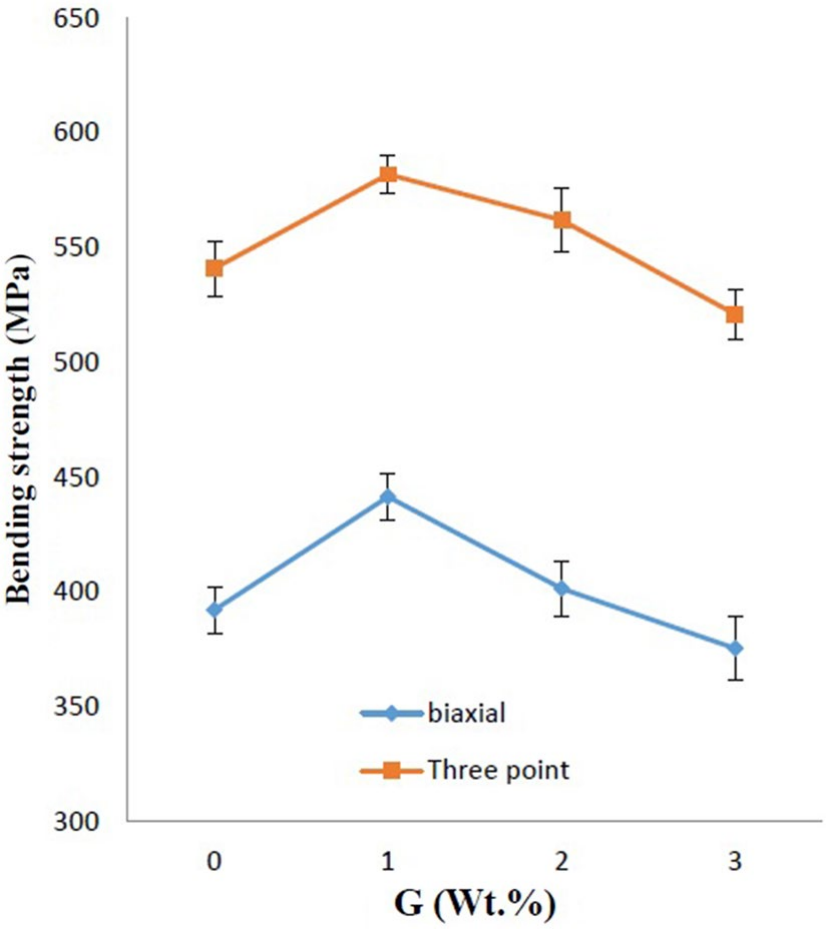
Comparison of three-point bending strength and biaxial bending strength.

Next, in addition to the three-point bending strength measurement method, the biaxial bending test (piston on three balls) was used to measure the strength of tablet-shaped samples. The graph of these changes for the samples containing 5 wt.% of nano β-SiC with different graphenes is shown in Fig. 9.

The results showed that the biaxial bending strength is lower than the three-point bending strength. Also, the maximum three-point bending strength of 582.01 MPa and biaxial bending strength of 441.56 MPa was obtained in the samples of 5 wt.% of nano β-SiC and 1 wt.% of graphene (5B1G). Studies have shown that in addition to the many advantages of using the biaxial bending strength, the results are very similar to the three-point bending strength.

## Conclusion

In this research, the effect of simultaneous addition of nano β-SiC and graphene on the mechanical properties of SiC ceramics was investigated. According to the results, adding 5 wt.% β-SiC along with 1 wt.% graphene improved the mechanical properties of solid-phase sintered SiC. The highest hardnesses of 28.03 GPa and 29.97 GPa were obtained in sample 5B with forces of 10 N and 1 N, respectively. The most-increased Relative density of 99.04%, Young’s modulus of 537.76 GPa, Fracture toughness of 5.73 MPa × m^1/2^, three-point bending strength of 582.01 MPa and biaxial bending strength of 441.56 MPa were obtained in 5B1G sample. Density and the formation of microstructure containing elongated and coaxial grains are among the factors affecting the improvement of mechanical properties of samples. It was also found that increasing the amount of additives leads to their agglomeration and reduced mechanical properties. Among the active mechanisms in improving the fracture toughness of this composite, crack deflection, branching and bridging, and the formation of cleavage steps can be considered. The type of fracture in optimal samples was also a combination of transgranular and intergranular ones. In addition, studies have shown that in addition to the many advantages of using the biaxial bending method, the results are very similar to the three-point bending strength.

## Data Availability

The datasets used and analyzed during the current study are available from the corresponding author on reasonable request.

## References

[1] Khodaei M, Yaghobizadeh O, Shahraki AA, Esmaeeli S (2019). Investigation of the effect of Al_2_O_3_–Y_2_O_3_–CaO (AYC) additives on sinterability, microstructure and mechanical properties of SiC matrix composites: A review. Int. J. Refract. Met. Hard Mater..

[2] Kultayeva S, Kim YW (2020). Mechanical, thermal, and electrical properties of pressureless sintered SiC–AlN ceramics. Ceram. Int..

[3] Kheyrinia L, Baharvandi HR, Ehsani N, Yaghobizadeh O (2019). Fabrication of SiC bodies by optimized gel-casting method. Int. J. Refract. Met. Hard Mater..

[4] Kim HM, Kim YW (2019). Low temperature pressureless sintering of silicon carbide ceramics with alumina–yttria–magnesia-calcia. J. Ceram. Soc. Jpn..

[5] Santos AC, Ribeiro S (2018). Liquid phase sintering and characterization of SiC ceramics. Ceram. Int..

[6] Ribeiro S, Gênova LA, Ribeiro GC, Oliveira MR, Bressiani AHA (2017). Effect of temperature and heating rate on the sintering performance of SiC-Al_2_O_3_-Dy_2_O_3_ and SiC-Al_2_O_3_-Yb_2_O_3_ systems. Ceram. Int..

[7] Seo YK, Eom JH, Kim YW (2018). Process-tolerant pressureless-sintered silicon carbide ceramics with alumina-yttria-calcia-strontia. J. Eur. Ceram. Soc..

[8] Razmjoo A, Baharvandi HR, Ehsani N (2022). The effect of graphene addition on the properties of SiC ceramics—A review. J. Aust. Ceram. Soc..

[9] Dehghani H, Khodaei M, Yaghobizadeh O, Ehsani N, Baharvandi HR, Alhosseini SHN, Javi H (2021). The effect of AlN-Y_2_O_3_ compound on properties of pressureless sintered SiC ceramics–A review. Int. J. Refract. Met. Hard Mater..

[10] Razmjoo A, Baharvandi HR, Ehsani N (2022). Pressureless sintering of SiC matrix composites reinforced with nano-β-SiC and grapheme. J. Korean Ceram. Soc..

[11] Khodaei M, Yaghobizadeh O, Baharvandi HR, Dashti A (2018). Effects of different sintering methods on the properties of SiC-TiC, SiC-TiB2 composites. Int. J. Refract. Met. Hard Mater..

[12] Bucevac D, Boskovic S, Matovic B, Krstic V (2010). Toughening of SiC matrix with in-situ created TiB2 particles. Ceram. Int..

[13] Forquin P, Rossiquet G, Zinszner JL, Erzar B (2018). Microstructure influence on the fragmentation properties of dense silicon carbides under impact. Mech. Mater..

[14] Zhan GD, Mitomo M, Tanaka H, Kim YW (2004). Effect of annealing conditions on microstructural development and phase transformation in silicon carbide. J. Am. Ceram. Soc..

[15] Noviyanto A, Yoon DH (2013). Metal oxide additives for the sintering of silicon carbide: Reactivity and densification. Curr. Appl. Phys..

[16] Noviyanto A, Yoon DH (2013). Rare-earth oxide additives for the sintering of silicon carbide. Diam. Relat. Mater..

[17] Jang SH, Kim YW, Kim KJ, Lee SJ, Lim KY (2015). Effects of Y_2_O_3_–RE_2_O_3_ (RE = Sm, Gd, Lu) additives on electrical and thermal properties of silicon carbide ceramics. J. Am. Ceram. Soc..

[18] Bahaaddini M, Baharvandi HR, Ehsani N, Khajehzadeh M, Tamadon A (2019). Pressureless sintering of LPS-SiC (SiC-Al_2_O_3_-Y_2_O_3_) composite in presence of the B4C additive. Ceram. Int..

[19] Ahmoye D, Bucevac D, Krstic VD (2018). Mechanical properties of reaction sintered SiC-TiC composite. Ceram. Int..

[20] Su B, Liu G, Huang Z, Liang H, Liu X, Chen Z (2015). The effect of in situ synthesized AlN on densification of SiC ceramics by pressureless sintering. Ceram. Int..

[21] Il Yun S, Youm MR, Nahm S, Park SW (2021). Fabrication and properties of macro-porous SiC using Al_2_O_3_–Y_2_O_3_–SiO_2_ as bonding additives. Ceram. Int..

[22] Malik R, Kim YH, Kim YW (2020). Effect of additive content on the mechanical and thermal properties of pressureless liquid-phase sintered SiC. J. Asian Ceram. Soc..

[23] Kim YW, Mitomo M, Zhan GD (1999). Mechanism of grain growth in liquid-phase-sintered β–SiC. J. Mater. Res..

[24] Nader M, Aldinger F, Hoffmann MJ (1999). Influence of the α/β-SiC phase transformation on microstructural development and mechanical properties of liquid phase sintered silicon carbide. J. Mater. Sci..

[25] Liu M, Yang Y, Wei Y, Li Y, Zhang H, Liu X, Huang Z (2019). Preparation of dense and high-purity SiC ceramics by pressureless solid-state-sintering. Ceram. Int..

[26] Magnani G, Sico G, Brentari A, Fabbri P (2014). Solid-state pressureless sintering of silicon carbide below 2000 °C. J. Eur. Ceram. Soc..

[27] Jana DC, Barick P, Saha BP (2018). Effect of sintering temperature on density and mechanical properties of solid-state sintered silicon carbide ceramics and evaluation of failure origin. J. Mater. Eng. Perform..

[28] Khodaei M, Yaghobizadeh O, Ehsani N, Baharvandi HR, Dashti A (2018). The effect of TiO_2_ additive on sinterability and properties of SiC-Al_2_O_3_-Y_2_O_3_ composite system. Ceram. Int..

[29] Liang H, Yao X, Liu X, Huang Z (2014). The effect of powder bed on the liquid phase sintering of α-SiC. Mater. Des..

[30] Liang H, Yao X, Zhang J, Liu X, Huang Z (2014). The effect of rare earth oxides on the pressureless liquid phase sintering of α-SiC. J. Eur. Ceram. Soc..

[31] Khodaei M, Yaghobizadeh O, Safavi SA, Ehsani N, Baharvandi HR, Esmaeeli S (2020). The effect of TiC additive with Al_2_O_3_-Y_2_O_3_ on the microstructure and mechanical properties of SiC matrix composites. Adv. Ceram. Prog..

[32] Khodaei M, Yaghobizadeh O, Ehsani N, Baharvandi HR, Bayati MB, Esmaeeli S, Javi H (2021). Improvement toughness of SiC ceramic by adding Cr_2_O_3_ and annealing process. J. Aust. Ceram. Soc..

[33] Feng D, Ren Q, Ru H, Wang W, Jiang Y, Ren S, Zhang C (2019). Effect of oxygen content on the sintering behaviour and mechanical properties of SiC ceramics. Ceram. Int..

[34] Liang H, Yao X, Huang Z, Zeng Y, Su B (2016). The relationship between microstructure and flexural strength of pressureless liquid phase sintered SiC ceramics oxidized at elevated temperatures. Ceram. Int..

[35] Khodaei M, Yaghobizadeh O, Naghavi Alhosseini SH, Esmaeeli S, Mousavi SR (2019). The effect of oxide, carbide, nitride and boride additives on properties of pressureless sintered SiC: A review. J. Eur. Ceram. Soc..

[36] Khodaei M, Yaghobizadeh O, Baharvandi HR, Esmaeeli S, Javi H (2020). The effect of Cr2O3 additions on sinterability and mechanical properties of liquid-phase sintered SiC ceramics. J. Alloys Compd..

[37] Khodaei M, Yaghobizadeh O, Baharvandi HR, Shahraki AA, Mohammadi H (2020). The effect of nano-TiO2 additions on the densification and mechanical properties of SiC-matrix composite. Ceram. Int..

[38] Miranzo P, López-Mir L, Román-Manso B, Belmonte M, Osendi MI, Ocal C (2016). Prominent local transport in silicon carbide composites containing in-situ synthesized three-dimensional graphene networks. J. Eur. Ceram. Soc..

[39] Huang Y, Jiang D, Zhang X, Liao Z, Huang Z (2018). Enhancing toughness and strength of SiC ceramics with reduced graphene oxide by HP sintering. J. Eur. Ceram. Soc..

[40] Chen C, Han X, Shen H, Tan Y, Zhang H, Qin Y, Peng S (2020). Preferentially oriented SiC/graphene composites for enhanced mechanical and thermal properties. Ceram. Int..

[41] Bódis E, Cora I, Balázsi C, Németh P, Károly Z (2017). Spark plasma sintering of graphene reinforced silicon carbide ceramics. Ceram. Int..

[42] Li Q, Zhang Y, Gong H, Sun H, Li T (2015). Effects of graphene on the thermal conductivity of pressureless-sintered SiC ceramics. Ceram. Int..

[43] Liu F, Wang M, Chen Y, Gao J, Ma T (2019). Mechanical properties and microstructure of reaction sintering SiC ceramics reinforced with graphene-based fillers. Appl. Phys. A.

[44] Li Q, Zhang Y, Gong H, Sun H, Li W, Ma L, Zhang Y (2016). Enhanced fracture toughness of pressureless-sintered SiC ceramics by addition of graphene. J. Mater. Sci. Technol..

[45] Guo X, Wang R, Zheng P, Lu Z, Yang H (2019). Pressureless sintering of multilayer graphene reinforced silicon carbide ceramics for mechanical seals. Adv. Appl. Ceram..

[46] Miura D, Ishida Y, Miyasaka T, Shinya A, Aoki H (2020). Reliability of different bending test methods for dental press ceramics. Materials.

[47] Mandal S, Seal A, Dalui SK, Dey AK, Ghatak S, Mukhopadhyay AK (2001). Mechanical characteristics of microwave sintered silicon carbide. Bull. Mater. Sci..

[48] Miura D, Miyasaka T, Aoki H, Aoyagi Y, Ishida Y (2017). Correlations among bending test methods for dental hard resins. Dent. Mater. J..

[49] Jin J, Takahashi H, Iwasaki N (2004). Effect of test method on flexural strength of recent dental ceramics. Dent. Mater. J..

[50] H.N. Yoshimura, Y. Zhou, H. Tanaka, Sintering OF 6H (α)-SiC AND 3C (β)-SiC commercial powders with B4C and C additives. Congr. Bras. Eng. E CIÊNCIA DOS Mater., 14, 03901–03912. https://www.ipen.br/biblioteca/cd/cbecimat/2000/Docs/TC102-011.pdf (Accessed 13 April 2022) (2000).

[51] Hilmas GE, Tien TY (1999). Effect of AlN and Al_2_O_3_ additions on the phase relationships and morphology of SiC part I compositions and properties. J. Mater. Sci..

[52] Zhou Y, Tanaka H, Otani S, Bando Y (1999). Low-Temperature pressureless sintering of alpha-SiC with Al4C3-B4C-C additions. J. Am. Ceram. Soc..

[53] Lim CS (2000). Effect of α-SiC on the microstructure and toughening of hot-pressed SiC-AlN solid solutions. J. Mater. Sci..

[54] Anstis GR, Chantikul P, Lawn BR, Marshall DB (1981). A critical evaluation of indentation techniques for measuring fracture toughness: I, direct crack measurements. J. Am. Ceram. Soc..

[55] Niihara K, Morena R, Hasselman DPH (1982). Evaluation ofK Ic of brittle solids by the indentation method with low crack-to-indent ratios. J. Mater. Sci. Lett..

[56] Cantarero A (2015). Raman scattering applied to materials science. Proc. Mater. Sci..

[57] Delobel F, Lemonnier S, Barraud É, Cambedouzou J (2019). Influence of sintering temperature and pressure on the 3C–6H transition of silicon carbide. J. Eur. Ceram. Soc..

[58] Wasyluk J, Perova TS, Kukushkin SA, Osipov AV, Feoktistov NA, Grudinkin SA (2010). Raman investigation of different polytypes in SiC thin films grown by solid-gas phase epitaxy on Si (111) and 6H-SiC substrates. Mater. Sci. Forum.

[59] Lorenzzi J, Zoulis G, Kim-Hak O, Jegenyes N, Carole D, Cauwet F, Juillaguet S, Ferro G, Camassel J (2010). Low doped 3C-SiC layers deposited by the vapour-liquid-solid mechanism on 6H-SiC substrates. Mater. Sci. Forum.

[60] Petrus M, Wozniak J, Cygan T, Kostecki M, Olszyna A (2019). The effect of the morphology of carbon used as a sintering aid on the mechanical properties of silicon carbide. Ceram. Int..

[61] Tanaka H, Sömiya S, Inomata Y (1991). Sintering of silicon carbide. Silicon Carbide Ceramics.

[62] She, J.H., Ueno, K. Effect of additive content on liquid-phase sintering on silicon carbide ceramics. Mater. Res. Bull., 34, 1629–1636. https://www.sciencedirect.com/science/article/pii/S0025540899001725 (Accessed 25 September 2019) (1999).

[63] Petrus M, Wozniak J, Cygan T, Adamczyk Cieslak B, Kostecki M (2017). Sintering behaviour of silicon carbide matrix composites reinforced with multilayer graphene. Ceram. Int..

[64] Sedlák R, KovalĿíková A, Girman V, Múdra E, Rutkowski P, Dubiel A, Dusza J (2017). Fracture characteristics of SiC/graphene platelet composites. J. Eur. Ceram. Soc..

[65] Nauyoks S, Wieligor M, Zerda TW, Balogh L, Ungar T, Stephens P (2009). Stress and dislocations in diamond-SiC composites sintered at high pressure, high temperature conditions. Compos. A Appl. Sci. Manuf..

[66] Zhou Y, Xiang H, Dai FZ (2019). Y5Si3C and Y3Si2C2: Theoretically predicted MAX phase like damage tolerant ceramics and promising interphase materials for SiCf/SiC composites. J. Mater. Sci. Technol..

[67] Awaji H, Choi SM, Yagi E (2002). Mechanisms of toughening and strengthening in ceramic-based nanocomposites. Mech. Mater..

[68] Choi SM, Awaji H (2004). Nanocomposites—A new material design concept. Sci. Technol. Adv. Mater..

[69] Moradkhani A, Baharvandi H (2018). Mechanical properties and fracture behavior of B4C-nano/micro SiC composites produced by pressureless sintering. Int. J. Refract. Met. Hard Mater..

[70] Llorente J, Román-Manso B, Miranzo P, Belmonte M (2016). Tribological performance under dry sliding conditions of graphene/silicon carbide composites. J. Eur. Ceram. Soc..

[71] Cordero ZC, Knight BE, Schuh CA (2016). Six decades of the Hall-Petch effect—A survey of grain-size strengthening studies on pure metals. Int. Mater. Rev..

